# Impact of HIV co-infection on plasma level of cytokines and chemokines of pulmonary tuberculosis patients

**DOI:** 10.1186/1471-2334-14-125

**Published:** 2014-03-04

**Authors:** Adane Mihret, Markos Abebe, Yonas Bekele, Abraham Aseffa, Gerhard Walzl, Rawleigh Howe

**Affiliations:** 1 Armauer Hansen Research Institute, Addis Ababa, Ethiopia; 2 Department of Microbiology, Immunology and Parasitology, School of Medicine, College of Health Sciences, Addis Ababa Univrsity, Addis Ababa, Ethiopia; 3 DST/NRF Centre of Excellence for Biomedical Tuberculosis Research and MRC Centre for Molecular and Cellular Biology, Division of Molecular Biology and Human Genetics, Faculty of Medicine and Health Sciences, Stellenbosch University, PO Box 19063, Francie van Zijl Drive, Tygerberg, 7505, South Africa

**Keywords:** Pulmonary tuberculosis, HIV, Cytokines and chemokines

## Abstract

**Background:**

The immunologic environment during HIV/*M. tuberculosis* co-infection is characterized by cytokine and chemokine irregularities that have been shown to increase immune activation, viral replication, and T cell dysfunction.

**Methods:**

We analysed *ex vivo* plasma samples from 17 HIV negative and 16 HIV pulmonary tuberculosis co infected cases using Luminex assay to see impact of HIV co-infection on plasma level of cytokines and chemokines of pulmonary tuberculosis patients before and after anti Tuberculosis treatment.

**Results:**

The median plasma level of IFN-γ, IL-4, MCP-3, MIP-1β and IP-10 was significantly different (P < 0.05) before and after treatment in HIV negative TB patients but not in HIV positive TB patients. There was no significant difference between HIV positive and HIV negative TB patients (P > 0.05) in the plasma level of any of the cytokines or chemokines before treatment and anti TB treatment did not change the level of any of the measured cytokines in HIV positive tuberculosis patients. The ratio of IFN-γ/IL-10 and IFN-γ/IL-4 showed a significant increase after treatment in HIV negative TB cases but not in HIV positive TB cases which might indicate prolonged impairment of immune response to TB in HIV positive TB patients as compared to HIV negative tuberculosis patients.

**Conclusions:**

HIV positive and HIV negative Tuberculosis patients display similar plasma cytokine and chemokine pattern. However, anti TB treatment significantly improves the Th1 cytokines and level of chemokines but does not restore the immune response in HIV positive individuals.

## Background

Tuberculosis (TB) and HIV/AIDS have proven to be a deadly and mutually reinforcing combination. In the absence of anti-retroviral therapy, HIV-infected individuals with latent tuberculosis infection have 5–10% annual risk of TB disease in contrast to 10% during life-time in HIV negative individuals [1]. A high risk of TB has been shown in early stages of the HIV disease, even in the presence of normal CD4+ cell counts [2]. The factors contributing to this increased risk are not clear, but may include a compromised response by CD4+ T cells, defective innate immunity and/or other elements of the host response against *Mycobacterium tuberculosis*[3,4].

Understanding what constitutes protective immunity to TB is critical for development of new diagnostics, treatment protocols and identifying vaccine candidates. In order to understand the immune profile of TB disease and infection, a number of parameters need to be considered. These include the HIV status with the majority of diagnostic tests not applicable for HIV positive patients. The cytokine response to TB antigens has been studied extensively but results have varied possibly due to differences in the genetic background of the study population, the type and length of antigen stimulation, the experimental protocol used and the sample type [5-7].

Although the pathogenesis of HIV-tuberculosis co-infection is not well understood, the immunologic environment during *M. tuberculosis* infection is characterized by cytokine and chemokine irregularities that are believed to increase T cell activation, enhance HIV replication and result in a dysfunctional immune response [8-12]. A reduction of Immune activation and HIV viral load after treatment of opportunistic infections other than TB is well recognized. Treatment of TB on the level of cytokines and chemokines among HIV-infected adults with tuberculosis is poorly characterized and effect of TB treatment on viral load and plasma cytokine response have been contradictory where some studies shown an increase pro-inflammatory immune response and decrease HIV viral load in response to TB therapy [9,13] while in other studies not [14-16]. Some studies also shown decrease in immune activation with no effect on viral load [17].

We recently described in a cross-sectional study that anti TB treatment changed the plasma level of cytokines and chemokines in HIV negative TB patients [18]. Here we used the same panel of cytokines to explore the hypothesis that there is a difference between the immune response before and after anti TB treatment in TB patients who are HIV positive compared to those who are HIV negative. We used direct unstimulated plasma samples assuming the sample represents the systemic activation in vivo and reflects the systemic immune response to see the immune response in TB cases who are HIV positive and HIV negative and for monitoring anti TB treatment outcome in both groups.

## Materials and methods

### Study subjects

We obtained ethical clearance from AHRI/ALERT Ethics Review Committee (P015/10) and National Research Ethics Review Committee (NRERC) (3.10/17/10). The participants voluntarily agreed to participate in the study and signed an informed consent form. In this study 33 individuals with active TB disease (17 HIV negative and 16 HIV positive) were recruited and followed from 4 health centers in Addis Ababa, Ethiopia. Diagnosis for TB was made by smear microscopy for AFB. All TB cases were new smear positive pulmonary TB cases and treated for 8 months with 2RHZE/6RE under DOTS and treatment adherence had been assessed by health extension workers. Effective anti TB therapy had been checked and confirmed by negative sputum acid fast bacillus at the end of the treatment.

All sputum samples from TB cases were cultured for mycobacteria. The presence of HIV infection was tested using rapid tests (Stat pack, KHP and Unigold as a tie breaker) as per the national guideline. In Ethiopia all patients attending TB clinics screened for sexually transmitted infections including HIV using a set of simple questions. As soon as HIV is identified in a TB patient, the patient is enrolled to HIV chronic care. The HIV care is delivered at the TB clinic for the duration of TB treatment or the patient may be referred to an HIV Chronic Care/ART clinic. The decision to initiate ART to TB patients is made by a trained clinician based on their CD 4 count. The first priority for HIV-positive TB patients is to initiate TB treatment, followed by cotrimoxazole and ART. HIV positive TB patients with profound immune-suppression (CD4 counts less than 50 cells cells/mm3) received ART immediately within the first 2 weeks of initiating TB treatment. Otherwise, timing of ART initiation is up to clinical judgment based on other signs of immunodeficiency indicating progression of HIV disease [19].

### Multiplex analysis

Blood sample was collected with heparin tubes twice at recruitment and after completion of anti TB treatment. Plasma was separated by centrifugation at 1000 rpm for 5 min and stored at -20 until the multiplex analysis was performed.

We used a 17plex kit (Epidermal Growth Factor (EGF), FRACTALKINE, Granulocyte Macrophage Colony Stimulating Factor (GM CSF), IFN-γ, IL-1, IL 10, IL-12, IL-17, IL-4, IL-7, IL-9, IFN-γ inducible protein (IP-10 /CXCL-10), Macrophage Chemo-attractant Protein 1 (MCP- 1/CXCL), MCP-3, Monocyte Inflammatory Protein 1 beta (MIP-1β), TNF and VEGF) from Millipore, Germany and multicytokine analysis was done using Luminex (Millipore, Germany) technology. The assays were performed according to the supplier’s instructions. Briefly, following pre wetting of plates, 50 μl of precombined beads from all the 17 individual cytokines or chemokines was added and washed twice after 1 hr incubation at room temperature. Plasma samples (25 μl) were diluted 1:1 with the kit serum matrix and added to the plate. The plate was shaken for 30 sec at 1000 RPM and then incubated for 1 hr on plate shaker (300 RPM) at room temperature. The plates were washed twice and 25 ul of detection antibody was added per well and incubated for 1 hr again on a plate shaker. Fifty micro liter of streptavidin-PE conjugate was added per well and incubated for 30 min at room temperature. Finally, the plate was washed 3 times and 150 μl of sheath fluid was added to each well and then the plate was read by Luminex machine (Millipore, Germany). Data was analysed using Luminex 100 IS software version 2.3.182. The Human Cytokine Quality Controls 1 and 2 and Assay buffer included in the kit were used as low and high concentration quality controls and background (Blank) respectively. Six non zero standard points ranging from 3.2 pg/ml to 10 000 pg/ml were assayed in duplicate to generate standard curve and the correlation coefficient (R^2^) was calculated in each experiment to see the linearity of the standard curve.

### Statistical analysis

The data were analyzed using Graph pad prism software, version 4.0. Data were log transformed to normalize their distribution. Nonparametric Mann–Whitney U tests were performed to check for the significance of the observed differences in each parameter in HIV positive and HIV negative TB cases.

## Results

We enrolled 33 subjects with active tuberculosis of which 16 were HIV positive and all were ART naïve with a mean CD4 count of 310 ± 23.9 cells/μL. The mean age of HIV positive TB patients and HIV negative TB patients was 29.6 ± 1.3 and 31.8 ± 1.2 respectively and 48.5% of the participants were females.

### Effect of HIV on plasma level of cytokines and chemokines on TB patients

**Figure 1:**
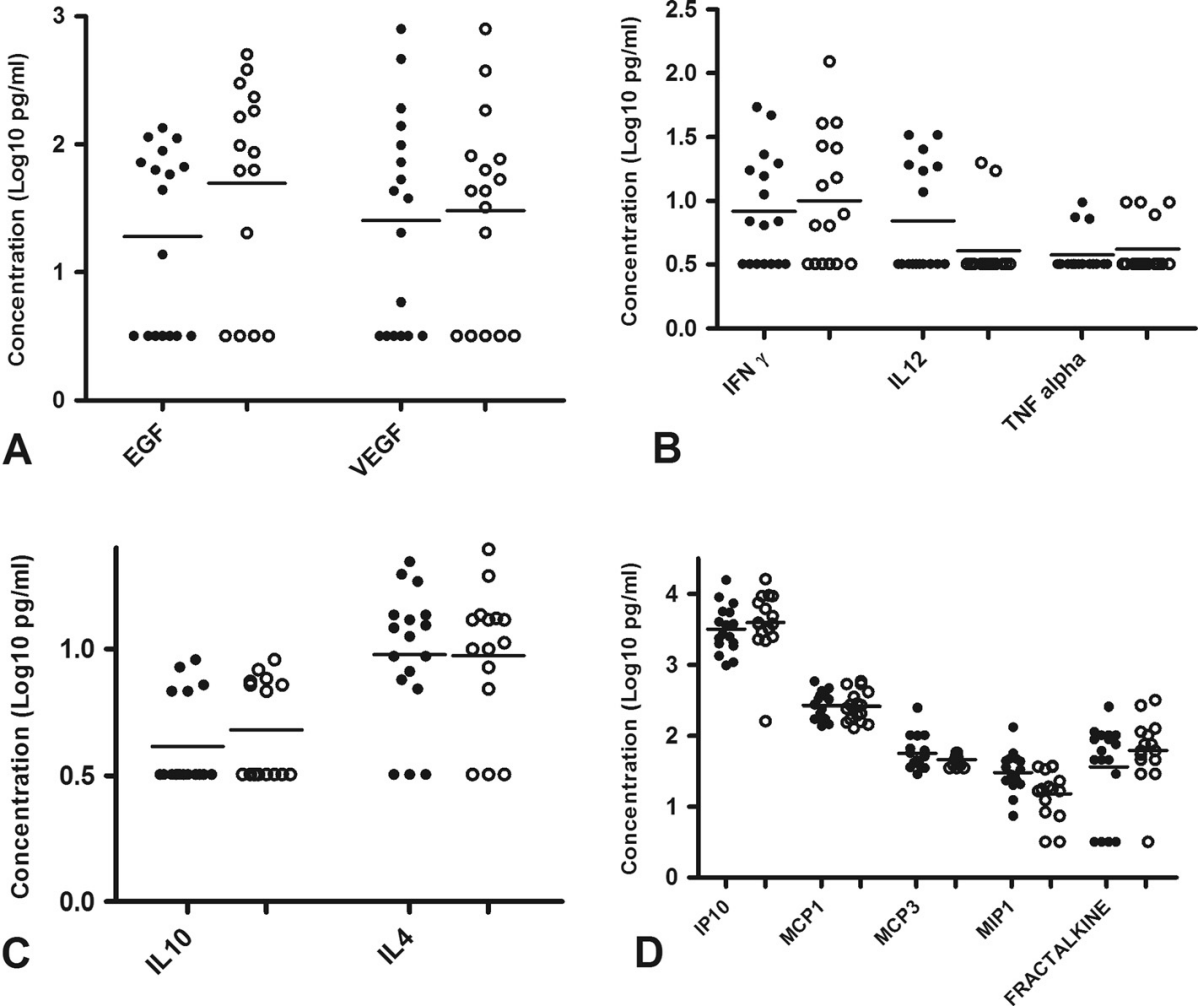
**Plasma cytokine and chemokine level in HIV negative and HIV positive TB cases.** Unstimulated plasma samples from HIV positive TB cases (n = 16) and HIV negative TB cases (n = 17) were assessed by multiplex cytokine analysis. **A)** Growth factors (EGF and VEGF) **B)** Pro-inflammatory cytokines (IFN-γ, IL 12(p40), and TNF) **C)** Anti-inflammatory cytokines (IL-10 and IL-4) and **D)** Chemokines (IP-10, MCP-1, MCP-3, MIP-1β and fractalkine). Horizontal lines indicate median of HIV negative (filled circles) and HIV positive TB cases (open circles). Data were analysed using nonparametric Mann–Whitney test with p-values indicating significant differences after transformation of data to Log10 values.

We found that the median plasma level of the cytokines and chemokines measured was not affected by HIV status although the HIV positive TB patients have a slightly higher level of most of the cytokines and chemokines (Figure 1).

### Impact of anti TB treatment on plasma level of cytokines and chemokines

**Figure 2:**
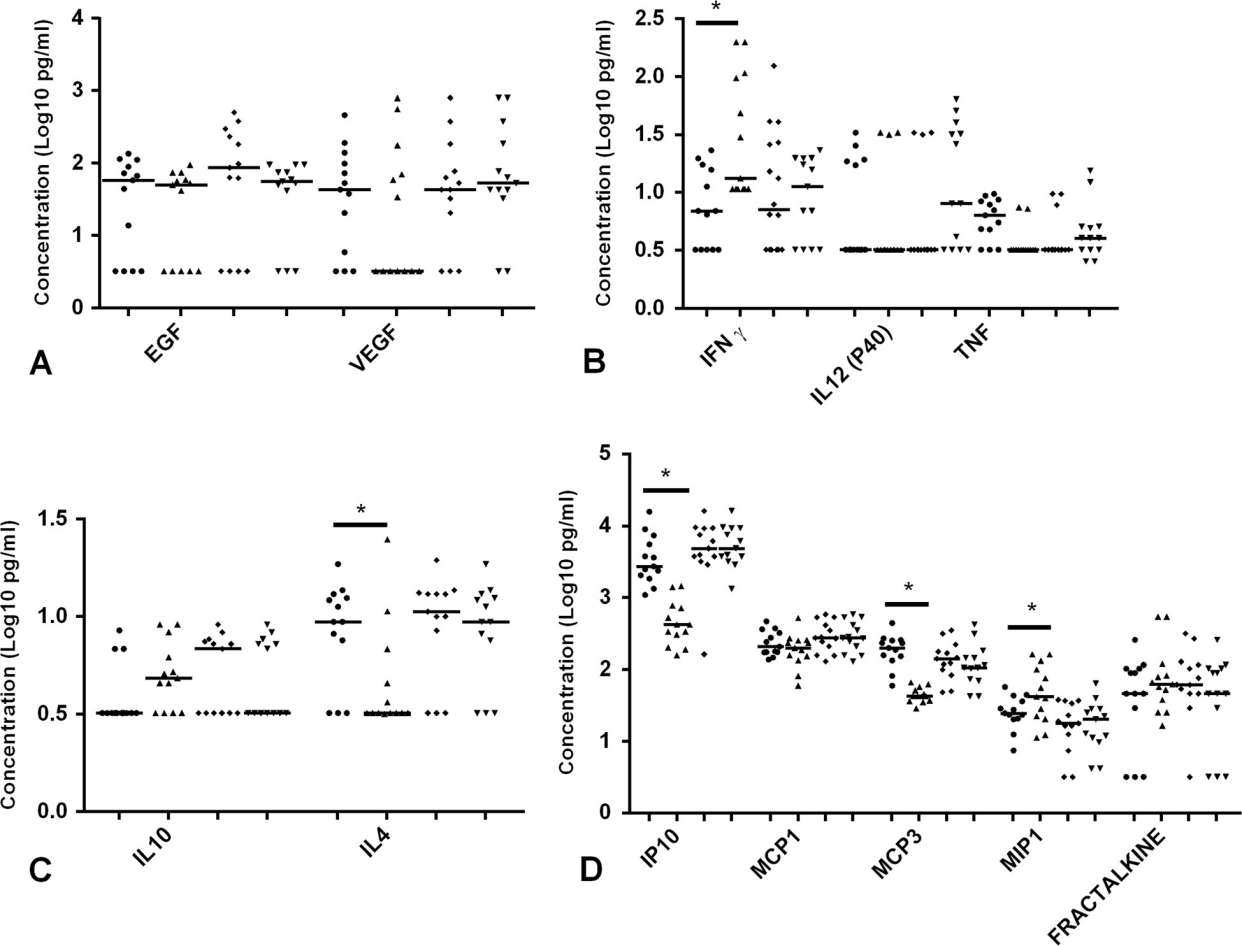
**Unstimulated plasma samples from HIV negative TB cases before treatment, after treatment (n = 17) and HIV positive TB cases before treatment and after treatment (n = 16) was assessed by multiplex cytokine analysis. A)** Growth factors (EGF and VEGF) **B)** Pro-inflammatory cytokines (IFN-γ, IL 12(p40), and TNF) **C)** Anti-inflammatory cytokines (IL-10 and IL-4) and **D)** Chemokines (IP-10, MCP-1, MCP-3, MIP-1β and fractalkine). Horizontal lines indicate median of HIV negative TB cases before treatment (filled circles), HIV negative TB cases after treatment (filled triangle), HIV positive TB cases before treatment (filled diamond) and HIV positive TB cases after treatment (filled inverted triangle). Data were analysed using nonparametric Mann–Whitney test with p-values indicating significant differences after transformation of data to Log10 values *p < 0.05.

We also measured the plasma level of the cytokines and chemokines after 8 month of effective anti-TB therapy to see if any of these cytokines and chemokines showed a difference among HIV positive and HIV negative TB patients as a result of treatment. We found that the median level of IFN-γ, IL-4, IP-10, MCP-3 and MIP-1β were statistically different (p < 0.05) after treatment in HIV negative TB patients but not in HIV positive TB patients (p > 0.05) (Figure 2). The median plasma level of IL-4 and IP-10 was significantly decreased whereas the level of IFN-γ, MCP-3 and MIP-1β significantly increased after treatment in HIV negative TB patients.

**Figure 3:**
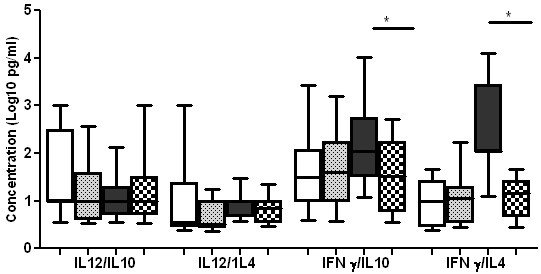
**Th1/Th2 ratios of cytokines of TB cases after anti TB treatment.** Box plots are shown with the horizontal line indicating median levels of TB cases and the lower and upper edge of each box indicate the 25th and 75th percentiles, respectively. Ratio of Th1/Th2 cytokines (IL-12/IL-10, IL-12/IL-4, IFN-γ/IL-10 and IFN-γ/IL-4) of TB patients who are HIV negative before treatment (white bars), TB patients who are HIV positive before treatment (Dotted bars), TB patients who are HIV negative after treatment (grey bars) and TB patients who are HIV positive after treatment negative (crossed bars). Data were analysed using nonparametric Mann–Whitney test with p-values indicating significant differences after transformation of data to Log10 values.

We also analysed the ratio of Th1 (IFN-γ and IL-12(p40)) and Th2 (IL-4 and IL-10) cytokines before and after treatment. None of the ratios of IFN-γ/IL-4, IFN-γ/IL-10, IL-12(p40)/IL-4 and IL-12(p40)/IL-10 were significantly different between TB patients who are HIV positive or HIV negative (Figure 2). Whereas the plasma concentration of IFN-γ/IL-4 and IFN-γ//IL-10 was significantly higher (p < 0.05) in HIV negative TB patients than HIV positive TB patients after treatment (Figure 3).

## Discussion

This study identifies differences in cytokine levels in TB patients with and without HIV and before and after anti TB treatment that may potentially plays a role in the protection or development of TB. We measured the plasma level of pro-inflammatory cytokines (IFN-γ, IL 12(p40), and TNF), Anti-inflammatory cytokines (IL-10 and IL-4), Growth factors (EGF and VEGF) and Chemokines (IP-10, MCP-1, MCP-3, MIP-1β and fractalkine).The main findings of this study were: (a) HIV co-infection does not seem to affect the median plasma level of any of the cytokine/chemokine in TB patients and there was no statistically significant difference between HIV positive and HIV negative TB patients; (b) The plasma level of IFN-γ, IL-4, IP-10, MCP-3 and MIP-1β significantly changed after completion of anti-TB treatment in HIV negative TB patients whereas in HIV positive TB patients none of the cytokines and chemokines showed significant difference after anti TB treatment; (c) The ratio of IFN-γ/IL-4 and IFN-γ/IL-10 were statistically different (p < 0.05) in HIV negative TB patients after treatment; and (d) The ratio of Th1/Th2 cytokines in HIV positive TB patients was not changed after treatment and it was significantly lower than HIV negative tuberculosis cases after anti TB treatment.

At baseline, TB + HIV + individuals displayed a similar plasma cytokine level when compared to TB + HIV- individuals. This could be due to both tuberculosis and HIV infection inducing immune activation in a similar pattern or due to the nature of HIV positive participants in this study where majority of them were at less advanced HIV infection stage as confirmed by their CD4 count. In our previous study we have shown that TB patients have a similar plasma cytokine and chemokine level with healthy household contacts after completion of anti TB treatment [18]. Different studies have shown that both tuberculosis and HIV infection can inhibit T cell effector functions, such as production of IFN-γ and interleukin-2, and co-infection is associated with more profound suppression of type-1 cytokine responses [10,20,21].

Longitudinal assessment of the plasma cytokine profile showed that out of the 17 cytokines measured, the plasma level of IFN-γ, IL-4, IP-10, MCP-3 and MIP-1β significantly changed after completion of anti-TB treatment in HIV negative TB patients whereas in HIV positive TB patients none of the cytokines and chemokines showed significant difference after anti TB treatment. The median plasma level of IL-4 and IP-10 was significantly decreased whereas the level of IFN-γ, MCP-3 and MIP-1β significantly increased after treatment. Because IFN-γ is a pro-inflammatory and IL-4 is an anti-inflammatory cytokine, it is reasonable to suggest that the increased concentration of IFN-γ and the reduced concentration of IL-4 might be due to the clearance of the actively multiplying bacteria and resolution of the disease following effective anti-tuberculosis treatment. The decrement of IP-10 and increment of MCP-1 and MIP-1β after treatment has been also reported by other studies [22-24]. IP-10 is involved in trafficking monocytes and activated Th1 cells to inflamed foci and the reduction of this chemokine could be due to resolution of inflammation as a result of anti TB treatment. The increased concentration of MCP-1 and MIP-1b is significant because of the key role these two cytokines play in the control of infection by attracting cells to the granuloma. In TB + HIV + patients the level of all of the cytokines and chemokines measured was not changed and this could be due to the ongoing inflammatory immune response.

The ratio of Th1/Th2 in HIV + and HIV- TB patients showed no difference before treatment whereas the ratio of IFN-γ/IL-4 and IFN-γ/IL-10 showed a significant difference before and after treatment in HIV negative TB cases where as none of the Th1/Th2 cytokines ratio showed a significant difference after treatment in HIV positive TB cases. The ratio of Th1/Th2 cytokines in HIV positive TB patients was lower than HIV negative TB cases. The higher level of IFN-γ/IL-4 and IFN-γ/IL-10 in HIV negative TB cases is expected where a Th1 type immune response dominates after clearance of the actively multiplying bacteria and resolution of the disease by the anti-tuberculosis treatment. In HIV positive TB patients anti TB treatment did not change the level of circulatory cytokines. It remains unclear whether tuberculosis treatment has a significant impact on other markers of HIV disease progression, such as T cell counts and changes in T cell subsets. Some studies reported that tuberculosis treatment increased CD4 T cell count among persons with HIV-tuberculosis co infection [13,25] whereas others indicated anti TB treatment didn’t show any change in absolute CD4 T cell count or restoration of the naive T cell population [26].

The lower ratio of IFN-γ/IL-4 and IFN-γ/IL-10 after anti TB treatment in HIV positive patients could be due to immune exhaustion by HIV co-infection with a possibility of future development of TB in HIV-infected individuals through down-regulation of IFN-γ and IL-12 and up-regulation of IL-4 and IL-10. The higher level of IL-10 and IL-4 in co-infected TB patients might be due to the immune exhaustion by HIV where the HIV-specific CD4 T cell response is inhibited through upregulation of IL-4 and IL-10*.* Previous studies suggested that the level of IL-10 is elevated in chronic HIV disease [27]. Moreover, lower IL-12 production by PBMC in response to TB antigens and lower levels of both Th1 and Th2 cytokines by PBMC correlate with increased susceptibility and development of TB in HIV-infected individuals and may be responsible for their increased susceptibility [28]. Moreover, a reduced ratio of IFN-γ/IL-4 might indicate increased disease severity in HIV positive TB patients as lower ratio of IFN-γ/IL-10 has been found to relate with increased disease severity in pulmonary and extrapulmonary TB [29].

The findings in this study however need further detailed analysis including (i) larger sample size, (ii) viral load and (iii) CD 4 count for HIV negative TB cases and after anti TB treatment for both groups to validate and confirm that the observed dysregulation of cytokine and chemokine production are indeed associated with increased susceptibility.

## Conclusions

HIV positive and HIV negative Tuberculosis patients display similar plasma cytokine and chemokine pattern. However, anti TB treatment significantly improves the Th1 cytokines and level of chemokines but does not restore the immune response in HIV positive individuals. This finding may suggest that in dually infected subjects, the HIV-related changes dominate the overall immunological picture and leads to dysregulation of cytokine and chemokine production.

## Competing interests

No conflict of interest.

## Authors’ contributions

AM involved in study design, data collection and analysis, data interpretation and drafted the manuscript. MA involved in analysis and write-up. YB involved in sample collection and laboratory work. AA, GW and RH were involved in study initiation, interpretation and write up of the manuscript. All authors read and approved the final manuscript.

## Pre-publication history

The pre-publication history for this paper can be accessed here:

http://www.biomedcentral.com/1471-2334/14/125/prepub
